# Improving the use of benzodiazepines-Is it possible? A non-systematic review of interventions tried in the last 20 years

**DOI:** 10.1186/1472-6963-10-321

**Published:** 2010-11-30

**Authors:** Alesha J Smith, Susan E Tett

**Affiliations:** 1 University of Queensland, School of Pharmacy, Brisbane, Queensland, 4072, Australia

## Abstract

**Background:**

Benzodiazepines are often used on a long term basis in the elderly to treat various psychological disorders including sleep disorders, some neurological disorders and anxiety. This is despite the risk of dependence, cognitive impairment, and falls and fractures. Guidelines, campaigns and prescribing restrictions have been used to raise awareness of potentially inappropriate use, however long term use of benzodiazepine and related compounds is currently increasing in Australia and worldwide. The objective of this paper is to explore interventions aimed at improving the prescribing and use of benzodiazepines in the last 20 years.

**Methods:**

Medline, EMBASE, PsychINFO, IPA were searched for the period 1987 to June 2007.

**Results:**

Thirty-two articles met the study eligibility criteria (interventions solely focusing on increasing appropriate prescribing and reducing long term use of benzodiazepines) and were appraised. Insufficient data were presented in these studies for systematic data aggregation and synthesis, hence critical appraisal was used to tabulate the studies and draw empirical conclusions. Three major intervention approaches were identified; education, audit and feedback, and alerts.

**Conclusions:**

Studies which used a multi-faceted approach had the largest and most sustained reductions in benzodiazepines use. It appears that support groups for patients, non-voluntary recruitment of GPs, and oral delivery of alerts or feedback may all improve the outcomes of interventions. The choice of outcome measures, delivery style of educational messages, and requests by GPs to stop benzodiazepines, either in a letter or face to face, showed no differences on the success rates of the intervention.

## Background

Benzodiazepines are used to treat various psychological disorders including sleep disorders, some neurological disorders and aspects of addiction and anxiety [1]. Utilization patterns show variation in the use of benzodiazepines over the last 2 decades [2,3]. During the 1990's there was much publicity around the harmful effects of long-term use of benzodiazepines in Australia, including new guidelines and efforts to increase community awareness [4-6]. This resulted in a decrease in prescribing of benzodiazepines, however since the end of these campaigns, use has been continually rising, with a 21% increase in utilisation by concession beneficiaries in Australia (elderly, over 65 years of age, and those with a low income or receiving benefits) from 2000 to 2006 [7].

It is known that prescribers and healthcare consumers need to be regularly reminded about reviewing long-term/chronic treatments and what the current best practice guidelines are[8,9]. This is especially relevant to benzodiazepines, as Australian guidelines state that benzodiazepines should be reserved for short term use only, be part of a broader treatment plan, and be prescribed at minimum doses[10,11]. This is due to the risks, including dependence, cognitive impairment, and falls and fractures[12-15]. Patients over the age of 65 years are particularly susceptible to these side effects due to accumulation and existing frailty[16,17].

Research has shown that these restrictions are not being implemented as the use of benzodiazepines continues to rise in Australia [7]. Whilst there have been a number of reviews exploring interventions to improve prescribing or change prescribing behavior [18,19], none have focused specifically on issues and interventions surrounding benzodiazepine use. It is therefore timely that a fresh approach is taken improve this problem.

The objective of this paper was to evaluate interventions aimed at changing the use of benzodiazepines over the last 20 years. This time period was chosen capture the interventions implemented during the 1980 s and 1990 s when there was increased focus on improving benzodiazepine use as well as the recent introduction of the newer benzodiazepine-related drugs (z-drugs). This evaluation will assist in determining a future effective strategy designed to increase the appropriateness of prescribing and use of benzodiazepine and related compounds.

## Methods

### Study Eligibility Criteria

Studies were included if they met the inclusion criteria, those which aimed to change benzodiazepine prescribing in any population either through education, using outreach or by raising awareness with consumers and/or health professionals, all study designs and locations were included for the period 1987-June 2007.

### Information Sources

A search was made of the following databases, Medline (via Ovid), EMBASE, PsychINFO and International Pharmaceutical Abstracts using the keywords (benzodiazepine(s) and anxi*) alone and in combination with intervention strategy(ies), educat* campaign(s), health intervention, intervention and utilis(z)ation. The search was limited to the English language. The reference lists of the retrieved articles and reviews were used to search for additional articles. A citation search was conducted and grey literature was also reviewed. The last search was completed in June 2007.

### Study Selection

The retrieved articles were screened by title (AS) any which clearly did not include an intervention about improving prescribing or use of benzodiazepines were excluded. Then abstracts for the remaining articles were scanned (AS) those studies which met the inclusion criteria were retrieved after abstract examination, for full text analysis.

Exclusion upon full text analysis was pre-determined for the following types of studies:

(a) studies only comparing 2 or more different dosage regimens or different ways of managing withdrawal (i.e. after the actual decision to change use had occurred).

(b) studies that did not distinctly report on the rates of prescribing or use of benzodiazepines (i.e. studies that intervened in overall psychotropic prescribing without differentiating benzodiazepines; or those not reporting rates of prescribing or use).

(c) studies that conducted an intervention in specific disease populations for whom benzodiazepines may be clinically indicated for continuous use (i.e. people with schizophrenia, epilepsy, or opioid drug users).

(d) reviews, double publications, animal studies and letters.

Any articles for which inclusion was uncertain were assessed by a second author (ST) until consensus was reached. All included studies were critically appraised and categorized into 1 of 3 groups; educational interventions, audit and feedback interventions, and alert interventions.

### Summary measures

For the purposes of this manuscript, an improvement in the use of benzodiazepines was defined by each study's pre-determined criteria. The results reported are in the same format as they were presented in the original article, this includes results presented in Table 1, 2 and 3, e.g. more appropriate use may be defined by the authors as a decrease in prescribing, perhaps, or a swap to short-acting benzodiazepine use rather than long acting in the elderly.

**Table 1:** Educational Interventions to decrease benzodiazepine use

Study	Location	Intervention design: *Educational Letter Interventions Targeting Consumers*	Study Design and Size	Result	Follow up
Bashir et al, 1994 [57]	London	Letters sent long term users (> 1 year) with a request to visit their GP and allocated to: 1 = informed about the correct use of BZ + self help book. 2 = control (no advice)	RCT 11 GP practices 109 patients.	Sig increase in the number of patients' that reduced BZ prescriptions in intervention group (18%) compared to control (5%).	6 months post intervention

Towle and Adams, 2006 [25]	Scotland	Letter sent to repeat BZ users by pharmacist to tell them that a step down program had been initiated and invited them to see their GP for a medication review + repeat BZ prescriptions were inactivated. Posters displayed in GP practice.	Convenience Sampling 1 practice, 206 patients.	73% collected their step down program. Decreased number of tablets prescribed by 64%. Only 23% of 369 patients stayed on a repeat script. (No statistical analysis).	3 years (end of the study)

Heather et al, 2004 [26]	UK	Long term BZ users (> 6 months) were allocated to: 1 = letter from GP asking them to consider cutting down their BZs. 2 = letter inviting participants to a consultation + leaflet and self help sleep book. 3 = control.	RCT 7 Practices, 299 patients.	Sig reduction in letter group (24%) vs. control (16%) and sig reduction in consult (22%) vs. control (16%). No sig difference between consultation and letter group for BZ decrease.	6 months post intervention

Cormack et al, 1989 [58]	UK	Long term BZ users (> 1 year) were allocated to: 1 = letter from GP advising patients to cut down BZ use. 2 = invitation to see the GP about cutting down BZ use. 3 = control.	CT 5 practices, 75 patients.	Sig reduction in BZ use in both the letter group and the consultation group compared to no change in control group (data not reported).	6 months to 1 year post intervention

Cormack et al, 1994 [23]	England	Long term BZ users (> 6 months) allocated to: 1 = letter from GP to recommending stopping BZs 2 = letter from GP + monthly advice sheets. 3 = control	CT 3 practices 209 patients.	30% reduction in BZ use by intervention groups (sig difference from control) No sig difference between interventions.	6 months post intervention

Gorgels et al, 2005 [59]	Netherlands	1 = discontinuation letter to long term users then approximately 3 months after the letter, an invitation for GP evaluation of BZ use. 2 = control.	RCT 30 practices 1707 patients (intervention) and 1821 (control).	Sig use reduction of BZ prescriptions in intervention group (24%) vs. control (5%). Sig reduction in BZ use in the intervention group for those who attended the GP evaluation (35%) vs. those who did not go (24%).	21 months post intervention

Morrison, 1990 [60]	UK	Long term BZ users (> 6 months) were informed that they should stop BZs by their GP. If agreed an individual plan (how to decrease and number of follow-up visits) was established.	Convenience Sampling 1 practice, 27/72 eligible patients agreed.	No new long term users started during study. 37.5% quit 33.3% reduced dose by >50% and 15% remained same dose or <50% reduction. No-one increased their dose during the study period.	1 year (end of study)

Onyett and Turpin, 1988 [28]	UK	Recruitment of long term users by notices. Asked to self-reduce BZs then all participants received a pamphlet and allocated to: 1 = group session. 2 = individual GP appointment.	Prospective cohort 18 patients.	59% reduction in dose (group) and 69% reduction in dose (GP meeting) but no sig difference between groups.	15 week post intervention

Brymer and Rusnell, 2000 [27]	Canada	Home assessment by nurse to determine if patients were substance dependent. Saw Geriatrician for medical review, who recommended a treatment plan (also sent to their GP). Were encouraged to join support/educational groups.	Observational study 55 elderly patients.	Significant reduction of BZ use (59%) between pre and post intervention.	6 months post intervention

**Study**	**Location**	**Intervention Design:** ***Educational Interventions Targeting GPs***	**Study Design and Size**	**Result**	**Follow up**

Zwar et al, 2000 [61]	Australia	1 = face to face 20 min educational visit by a GP focusing on the management of long term users of BZs + guidelines + leaflets on relaxation techniques for patients. 2 = control.	RCT 157 GP registrars	Sig decrease in overall BZs use in both groups (0.6 per 100 encounters for both group), however no difference between control and intervention groups.	3 practice surveys - 6 monthly

Smith et al, 1998 [30]	Washington, USA	1 = mailed intervention package (guidelines, letter, prescriber-specific profile, patient profile) for prescribers of over users (1 tab per day >1 year) 2 = control.	RCT GPs or pharmacist of 222 over users	Sig decrease in BZ prescribing/dose for the intervention group (27.6%) compared to control (8.5%).	3 months post intervention

Holm, 1990 [32]	Aarhus, Denmark	1 = invite to a meeting on correct use of hypnotics/sedatives + educational material given at the meeting. 2 = mailed information on correct use and feedback on their prescribing rate compared with others. 3 = control.	RCT 356 GPs (245 practices)	Sig decrease (-53) in DDD/1000 pat/week between pre and post intervention. No difference between groups 1 and 2 but sig difference between groups 2 and 3 (2 prescribed more).	1-2 months post intervention

De Burgh et al, 1995 [29]	NSW, Australia	1 = 20 min educational visit to GPs. Educational material left (management guidelines, review cards for long term users). Offered access to sleep aids. Recommended to review 5 patients and received a follow-up phone call. 2 = control.	RCT 286 GPs	An overall decrease in BZ prescribing (23.7%). No sig difference between intervention and control for reduction rate of BZ. Sig decrease between intervention (72% decrease) and control (13% decrease) for rate of new BZ scripts for insomnia.	Approx 6 months post intervention

Midlov et al, 2006 [62]	Skane, Sweden	1 = two educational visits from pharmacist and GP focusing on effects of medium and long acting BZs in the elderly. 2 = control (received intervention after the study).	RCT 15 GPs (8 in intervention, 7 in control)	Sig decreases in all BZ prescribing (26.63%) and 25.8% decrease of long and medium acting BZs vs. control.	1 year post intervention

Berings et al, 1994 [31]	East and West Flanders, Belgium	1 = educational advertisement like mailings (with slogan) + educational visit. 2 = mailings only 3 = control	RCT 128 physicians	Sig decrease for whole sample pre to post intervention. Sig decrease in BZ prescriptions between intervention 1 (24%) and control (3%) and intervention 2 (14%) and control and intervention 1 and 2.	4 weeks post intervention

**Study**	**Location**	**Intervention Design: *Educational Interventions Targeting LTC***	**Study Design and Size**	**Result**	**Follow up**

Hagen et al, 2005 [63]	Alberta	1 = Algorithm on non-pharmacological approaches for agitation. Education based on algorithm to nurses, pharmacists, or family members. 30 min education session for GP. 2 = Control	CT 24 LTC facilities, 12 (intervention) and 12 (control)	BZ use in both the control and the intervention increased post intervention (Sig increase for control only). At 6 months post intervention total BZ use sig higher in control vs. intervention.	Every 2 months until 6 months post intervention

Avorn et al, 1992 [64]	Massachusetts	1 = physicians received 3 × drug advertisement like summaries of literature about geriatric medicine, psychopharmacology + 3 face to face visits to each doc by pharmacist. 4 training sessions for nurses on alternatives to psychoactive drugs + ADRs. 2 = Control	MPR 12 LTC facilities 6 (intervention) and 6 (control) both groups were matched	Sig difference in the % change to more appropriate BZs in intervention (64%) vs. control (4%). E.g. long acting to short acting.	30 days post intervention

Schmidt et al, 1998 [65]	Sweden	1 = Monthly meeting for 12 months led by pharmacist and included physician, and nurses. Each patient's medications were reviewed. 2 = Control	RCT 33 nursing homes (15 intervention, 18 control)	Sig increase (from baseline) in prescribing of appropriate hypnotics (+70%) and anxiolytics (+ 50%) in intervention group. No sig difference in control group	1 month post intervention

Gilbert et al, 1993 [36]	Adelaide, Australia	1 = letter to residents inviting participation in relaxation groups (8 × 40 min) audio tape of relaxation for practice and information about sleep & anxiety medications. Nurses received a seminar on dealing with BZ withdrawal. Doctors received letter of progress. 2 = Control	Prospective cohort 2 LTC facilities	There was a sig decrease in the % of BZs users' from baseline (70%) to post intervention (35%). No change in control	12 weeks after baseline

BZ = benzodiazepines, GP = General Practitioner, LTC = long term care, Sig = statistically significant (p < 0.05), tab = tablet, ADRs = Adverse Drug Reactions, DDD/1000 pats/week = the number of defined daily doses dispensed per 1000 patients per week, RCT = Randomized controlled trial, CT = Controlled trial, MPR = Matched pair randomization

**Table 2:** Audit and Feedback Interventions to decrease benzodiazepine use

Study	Location	Intervention Design: *Audit and Feedback Interventions targeting GPs*	Study Design and Size	Result	Follow up
Baker et al, 1997 [66]	Leicester, UK	Audit on all long-term users (> 4 weeks) in the medical centre then GPs received either: 1 = feedback on prescribing practices + criteria for the management of long term BZ users. 2 = feedback + criteria + reminder cards for patient files.	RT 18 practices patients = 2409 long term BZ users	Both groups changed after intervention with respect to levels of compliance to criteria. 8.2% of patients were stopped and 1.3% were decreasing BZs. No difference between groups.	2^nd ^audit completed 1 year post intervention

Holden et al, 1994 [67]	Liverpool, Southport - UK	Audit of BZ use + GPs invited to 2 meetings on auditing BZ use in general practice. Individual practices determined their own BZ policy for prescribing and reducing use.	Observational 15 practices, 3234 patients	Overall reduction of 16%. Sig reduction in those <65 (25%) compared to those >65 = 12%.	2^nd ^audit at 8 months (end of study)

Pimlott et al, 2003 [68]	Canada - Ontario	1 = audit and feedback on GPs prescribing of BZs compared to peers and best practice + information sheet on BZs every 2 months for 6 months. 2 = Control group had the same intervention for antihypertensives.	RCT 168 GPs (intervention) 206 GPs (control)	No sig decrease in BZ prescribing and no sig difference between intervention and control groups.	6 months post intervention

**Study**	**Location**	**Intervention Design: *Audit and Feedback Targeting LTC***	**Study Design and Size**	**Result**	**Follow up**

McClaugherty, 1997 [69]	Texas	LTC pharmacist audited BZ use + gave feedback to nurses and doctors. Nurses were given sleep promoting guidelines. OT's & physio's were encouraged to increase activities for those who couldn't sleep.	Quasi-Experimental 10 Nursing Homes, 3 Texas counties	% of patients prescribed routine BZ decreased from 4.5% (baseline) to 1.6% (post intervention). % of patients prescribed BZ on an as needed basis increased from 7.9% (baseline) to 9.3% (post intervention)	3 months post intervention

Gill et al, 2001 [70]	Ontario, Canada	Review of patients chart + a letter was sent to the treating doctor if inappropriate e.g. long acting BZ explaining why medication was inappropriate and suggestions for alternative therapy.	Quasi-Experimental 1 LTC facility, 450 Patients	37.9% of inappropriate medications were withdrawn or changed after the letter.	2 months after follow-up letters

Elliot et al, 2001 [37]	Australia	Audit and 1 h meeting = feedback to all staff on prescribing compared to other hospitals and review of literature + posters in wards	Quasi-Experimental 9 hospitals (6 aged care 3 medical wards)	No sig reduction in BZ use. Sig increase in appropriate prescribing at 8 week (22%) and 6 months (30%) post intervention.	4-8 weeks (all) and 6 months (for 3 hospitals only) post intervention

**Study**	**Location**	**Intervention Design:** ***Audit and Feedback + Education Targeting LTC***	**Study Design and Size**	**Result**	**Follow up**

Roberts et al, 2001 [34]	QLD + NSW, Australia	1 = 11 hrs of problem based education session for nurses + wall charts, bulletins, telephone, visits. Written drug review for 500 selected patients. Report on review placed in patient's records and available to the GPs. 2 = Control	RCT 52 nursing homes, 13 (intervention), 39 (control).	Sig difference in the reduction of BZs between intervention (decreased 597 items/year/1000 residents) and control (increased 278 items/year/1000 residents).	12 months (end of study)

Batty et al, 2001 [35]	England/ Wales	Audit then: 1 = lecture to staff on literature review on appropriate prescribing of BZs. Feedback on prescribing compared to another hospitals. 2 = Bulletin (2 sided A4) with same information as lecture. 3 = Control	RCT Elderly inpatients at 17 hospitals (6 lecture, 4 bulletin, 7 control)	No sig change in any group but verbal group increased appropriate prescribing by 15%, bulletin decreased appropriate prescribing (9%) and control remained the same.	4-6 weeks post intervention

Eide and Schjott, 2001 [33]	Norway	1 = Audit of BZ use, feedback to staff (reports and a presentation). Academic education to all staff by pharmacist, consisting of 6 simple rules for the use of hypnotics (data collected in 1995 and 2000). 2 = Control (data collected in 2000 only)	CT 10 LTC Facilities, 5 (intervention) and 5 (control)	Sig dif in the % of patients use BZS in control (44%) compared to intervention (24%) post intervention. Sig higher dose of BZs in intervention group in 2000 (60%) compared to 1995 (38%).	5 years post intervention

Crotty et al, 2004 [71]	Adelaide, Australia	Audit then: 1 = GP received education and guidelines and audit of use. Nurses received education in behaviour management and all staff received education on reducing psychotropic medication use 2 = control	MRP 20 LTC facilites, 10 (intervention) and 10 (control)	No sig reduction in BZ use (6.3%, intervention, 0% control), no significant decrease in long acting BZs (2.8% intervention and 0.9% control) and no sig difference in BZ being prescribed on a as needed basis (4% intervention and 1% control)	2^nd ^audit was at 7 months (end of study)


BZ = benzodiazepine, GP = General Practitioner, Av = Average, Sig = statistically significant (p < 0.05), LTC = long term care, OT's = Occupational Therapists, RCT = Randomized controlled trial, CT = Controlled trial, RT = randomized trial, MPR = Matched pair randomisation

**Table 3:** Alert Interventions to decrease benzodiazepine use

Study	Location	Intervention Design: *Alert Interventions targeted at GPs*	Study Design and Size	Result	Follow up
Simon et al, 2006 [72]	USA - Oregon & Washington	1 = age Specific (> 65 years) alert for long acting BZs. 2 = alerts + academic detailing (group education + follow-up letter).	Cluster randomized trial 239 clinicians	No sig decrease in prescribing to elderly. No difference between alerts (decrease of 3.0 dispensed medications per 10,000 members) and alerts + academic detailing (decrease of 19.7 dispensed medications per 10,000)	18 months post intervention

Monane et al, 1998 [73]	America	1 = age specific (> 65 years) alert system at pharmacy (mail order and retail). If medication is deemed to be inappropriate then conversation between pharmacist and prescriber occurred. 2 = control.	Population based cohort 2.3 million people >65 years filled a script during study period	Sig difference between intervention (40% of cases) and control (2%) for the change of prescriptions for long acting BZs. Sig difference between control (2%) and intervention (25%) for change to prescriptions of short acting BZs that exceeded the maximum daily dose.	1 year study (measured total number of changes over 1 year)

BZ = benzodiazepine, Sig = statistically significant (p < 0.05), GP = General Practitioner

### Synthesis of results

Using the above summary measures, it is difficult to quantify 'success' or to quantitatively compare improvement between the studies due to the large variation in design and measurement. One of the major barriers is the definition of appropriateness of use for benzodiazepines used by each different study, and therefore what is meant by an improvement of use. This has been defined in a number of ways; some studies used criteria, such as Beers' Criteria [20], others just using quantities of benzodiazepines to ascertain whether recipients of benzodiazepines should have been receiving these medicines. Beers' Criteria and similar indicators are developed from populations, and it is difficult to apply these in a overarching fashion, without allowing for individual clinical need, so determining 'appropriate/inappropriate' based on these rigid criteria is often controversial [21,22]. Other studies simply indicated that an 'improvement' was a decrease in overall use of benzodiazepines [23]. Due to the variation in reporting and the lack of statistical analysis in many studies, this review has not included a meta analysis, instead the studies have been tabled so that the general attributes and the design of the studies can be compared, critiqued and discussed. It is from these critiques that factors influencing success or otherwise can be determined, and lessons about future study design learnt.

### Risk of Bias

Some intervention studies about benzodiazepines may have been excluded from this commentary due to the use of the selected key words, however it was difficult to be more general than the terms selected without generating a totally non-specific (and much larger) database of possible articles. There may be a publication bias, as studies are known to be less reported if they generate negative results (or 'no change') with non-completion of the intervention or write up more likely.

## Results

**Figure 1:**
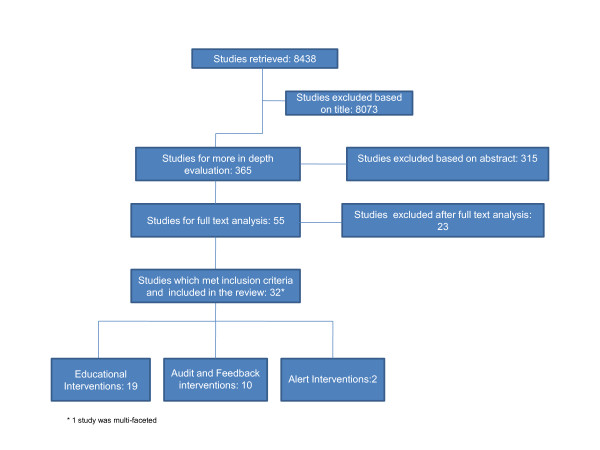
**Flow Chart of studies retrieved and included in the review**.

The search of the databases returned 8437 studies (Figure 1). Of these, 32 studies fulfilled all the pre-determined inclusion criteria and could be categorized as described above. For each study the data were extracted for the following characteristics; location of the intervention, intervention design, study type, participant numbers, intervention follow-up time and study outcomes. Comparisons of these characteristics are described below.

### Educational Interventions

The 19 studies (summarized in Table 1) have been targeted at 3 different groups; those aimed at GPs, at consumers and at long term care facilities.

### Audit and Feedback Interventions

The 10 studies (summarized in Table 2) were classified into 2 categories, those aimed at GPs and those aimed at long term care facilities. A sub category was also identified within the interventions aimed at long term care facilities, those which include education in addition to audit and feedback.

### Alert Interventions

These 2 studies (summarized in Table 3) were only targeted at GPs.

The final study included was a multifaceted intervention which used an educational approach to target GPs, pharmacists and consumers in a rural region of South Australia. A 19% reduction in benzodiazepine use was maintained 2 years after the intervention finished [24].

The critiques of the 32 studies (Table 1, 2 and 3) are discussed below.

### Education

#### Targeted at Consumers

Consumer focused educational intervention specifically targeted individual long term benzodiazepine users rather than an overall public health approach or community awareness campaigns and used mail-outs as the main approach. In all of these studies, long term benzodiazepine users were sent letters from a GP, or in one case from a pharmacist [25]. The letters asked that patients cut back on the benzodiazepines and gave basic advice on how to do this (e.g. gradually reducing use over a period of weeks). All of these studies saw a similar dose reduction (22% - 30%). Studies which invited participants to visit a GP, during which consultation they were asked to reduce their benzodiazepine dose face to face, resulted in significant decreases in benzodiazepine doses compared to the control but did not significantly differ to those who received only the letter [23,26]. This demonstrates that a simple bulk mail-out to identified individuals approach appears to be just as effective in improving use of benzodiazepines, and may have advantages compared to the more time consuming, and expensive approach of educational sessions or GP consultations.

Educational and support group sessions were also used as an educational method. The studies using group sessions did see larger reductions (69% and 59% dose reduction [27,28] respectively) in benzodiazepine use compared to the mail-out studies but neither of these studies used a control group so conclusions are difficult to draw.

#### Targeted at GPs

Education for GPs included packages containing benzodiazepine prescribing guidelines, face to face visits and 'promotional' material with key messages.

When compared to control, 50% of the educational interventions targeted at GPs had significant decreased benzodiazepine use and 17% (1 study, [29]) which did not reduce overall use of benzodiazepines did significantly decrease the number of new patients being prescribed benzodiazepines.

The study [30] that saw the largest reduction in benzodiazepine use differed from the other GP targeted educational studies as it did not rely upon voluntary participation. Any GP who prescribed to a Washington State Medicaid beneficiary deemed to be a "high utilizer" of benzodiazepines was sent the intervention package (see Table 1 for further details). In the other studies the voluntary recruitment design may have resulted in the participation of GPs who prescribed benzodiazepines more rationally (pre-intervention) than non-participating GPs. If this occurred, smaller changes between intervention (pre and post) and control groups would be seen, and this selection bias could be influencing the conclusions.

Differences in the delivery of the education (mail vs. personal visit) had varying effects on the "success" of the studies. For example, Smith et al, 1998 (the study with the largest decrease in benzodiazepine use) delivered education via mail-out. This success is supported by a study which had a mail-out and educational visit intervention arms [31]. They found a significant difference in the decrease of benzodiazepine use between control and both the educational visit (24%) and the mail out (14%) arms and between the two interventions. However Holm,1990 found no significant difference between the education visit and the mail-out arms of their intervention [32].

#### Targeted at long term care facilities

The educational tools used in long term care facilities varied from prescribing algorithms and bulletins to staff meetings and lectures.

Educational interventions delivered to long term care facilities differed compared to those delivered to GPs and consumers in two ways. Firstly, some studies included audit and feedback in addition to education [33-35] and all of the studies were multi faceted (the education was directed at a variety of people, including nurses, GPs, family members, pharmacists) rather than just targeting GPs. This multifaceted approach could in some part be responsible for the larger percentage of studies which either decreased use or reported improved prescribing of benzodiazepine in this setting when compared to the GP targeted studies.

The majority of the long term care facility educational studies used pharmacists to deliver the "educational message" to staff, (one study did not specify [34] and 1 used a psychologist [36]). Of the 5 pharmacist led studies two were unsuccessful in either decreasing benzodiazepine use or increasing appropriate prescribing suggesting that, in this context, the type of health professional delivering the education does not determine the success of the intervention.

### Audit and feedback

The audit and feedback studies which targeted long term care facilities involved more than 1 health professional e.g. doctors, nurses, physiotherapists and pharmacists whereas the GP targeted interventions only involved the GPs. This may be responsible for the difference in results between the 2 groups, as the audit and feedback studies in long term care facilities all improved appropriateness of prescribing (as defined by each study) but only 1 of the 3 GP studies achieved this same success. Audit and feedback may be resisted by some prescribers, which may explain perhaps why better results for benzodiazepine prescribing were obtained in long term care facility audits, where multiple different health practitioners were targeted compared to the audits of individual GPs alone.

Another factor that appears to affect the outcome of the interventions is how the feedback was delivered. All studies which successfully achieved their aim (reducing or improving use of benzodiazepines) gave feedback orally, either in a meeting or individual consultations instead of just mailing.

Both success and failure to improve benzodiazepine prescribing was seen in both the audit and feedback intervention group [37-39] and the audit and feedback in conjunction with education group [33-35,40]. It can therefore not be determined if education in addition to audit and feedback interventions improves the outcome of the study.

### Alerts

The GP targeted decision support tool alerted GPs when they were either prescribing a long acting benzodiazepine to a patient over 65 or initiating a patient on a benzodiazepine. This intervention found no difference between intervention and control when measuring the change in the target medications per 10,000 patients per quarter. This is not surprising as GPs are renowned for turning the electronic reminders/alerts off or ignoring all alerts that appear [41].

The second alert system was run through the pharmacy, where the pharmacist contacted the GP to discuss the prescription if an alert appeared. This study did see a significant increase in the appropriate prescribing of benzodiazepines (as determined by an independent advisory board and the use of Beers Criteria) [20]. An improvement was defined as a decrease in number of benzodiazepine prescriptions which, a) exceeded therapeutic dose, b) prescribed to the over 65 s and c) contained long acting benzodiazepines.

With both studies having a similar definition of "appropriate", the difference here appears to be the pharmacist involvement. It has been suggested that community pharmacists are an underused resources in prescribing studies [42], therefore it may be an appropriate time to incorporate pharmacists into future studies, as they are fast becoming one of the most utilized health professionals in many countries [43].

## Discussion

### Summary of Evidence

This investigation solely focuses on interventions designed to improve prescribing and/or use of benzodiazepines and related drugs in the last 20 years and has demonstrated that many different interventions strategies are used worldwide, with varying success. The most successful interventions to improve prescribing and use of benzodiazepines were those which are multi-faceted, targeting a number of groups (prescribers as well as consumers).

### Limitations

This study may be limited by the fact that this was not a complete systematic review and there are insufficient data available to conduct a meta-analysis on the effect size of any specific intervention, hence bias in interpretation may have been introduced by the non-quantitative summary techniques.

### Knowledge Translation

The points below outline some aspects of the study designs that may enhance the success of any future intervention.

Points which appear to enhance success of an intervention

• Advise individual patients to stop/cut back on their benzodiazepines (either via letter or face-to-face). A follow-up consultation may improve the success for individual patients.

• Encourage consumer support groups, to enhance self-withdrawal of benzodiazepines by patients

• Encourage inter-professional approach e.g. pharmacists alerting prescribers of potentially inappropriate prescribing of benzodiazepines or nurses regularly contacting/providing information to patients who are trying to cut back on their benzodiazepines

• Use oral feedback rather than written in audit and feedback studies to GPs; consider group audit, such as through long term care facilities, rather than audit of individuals

• Educate all long term care staff about alternative methods to treat insomnia and cutting back on the use of benzodiazepines

## Conclusions

One of the key success factors noted with all intervention approaches was the use of a multi-target design where a number of audiences or approaches were used; this may be why the consumer interventions worked well, as essentially they had two target audiences, the consumer and the GP or health professional involved in the study. This also agrees with findings from other therapeutic areas, for example a mass media campaign around appropriate use of antimicrobials and student taught programs for reducing antibiotic use during colds or flu [9,44,45]. The one distinctly multi-faceted study in this review of benzodiazepine interventions also saw a sustained reduction (19%) in benzodiazepine use when all health professionals and the community were educated [24]. Conducting multi-faceted interventions can often be labor intensive, expensive and difficult to administer on a large scale basis and therefore can usually only be maintained for a limited time. Future interventions which use delivery methods such as email or websites may help to ameliorate some of these financial or 'reach' problems. This commentary has suggested that the use of support/educational groups for consumers may improve the 'success' of an intervention, this could easily be administered using social networking technologies.

Whilst it is known that many healthcare providers have resisted using information technology due to privacy and infrastructure issues [46,47], many pharmacies, and medical centres now use the internet and email regularly and frequently [48-50]. Future investigation and validation into the effectiveness of electronic delivery method to a number of target groups could be beneficial, as varying results have been seen [51-54]. Studies which incorporate newer information technology may lead to more sustained changes as they are simple to administer, and can have a large scale impact by inexpensively involving a variety of health professionals and/or consumers [55,56].

## Competing interests

The authors declare that they have no competing interests.

## Authors' contributions

AS led the design and conduct of the study; interpretation of the results, and manuscript development. ST contributed to the interpretation of the results and manuscript development. All authors approved the final manuscript.

## Pre-publication history

The pre-publication history for this paper can be accessed here:

http://www.biomedcentral.com/1472-6963/10/321/prepub
